# Supplementary figures and images for: Rapid diagnosis of skin and soft tissue melioidosis in children
Source: PLoS Negl Trop Dis. 2026 Feb 3;20(2):e0013962. doi: 10.1371/journal.pntd.0013962 (PMC12880741; doi:10.1371/journal.pntd.0013962)

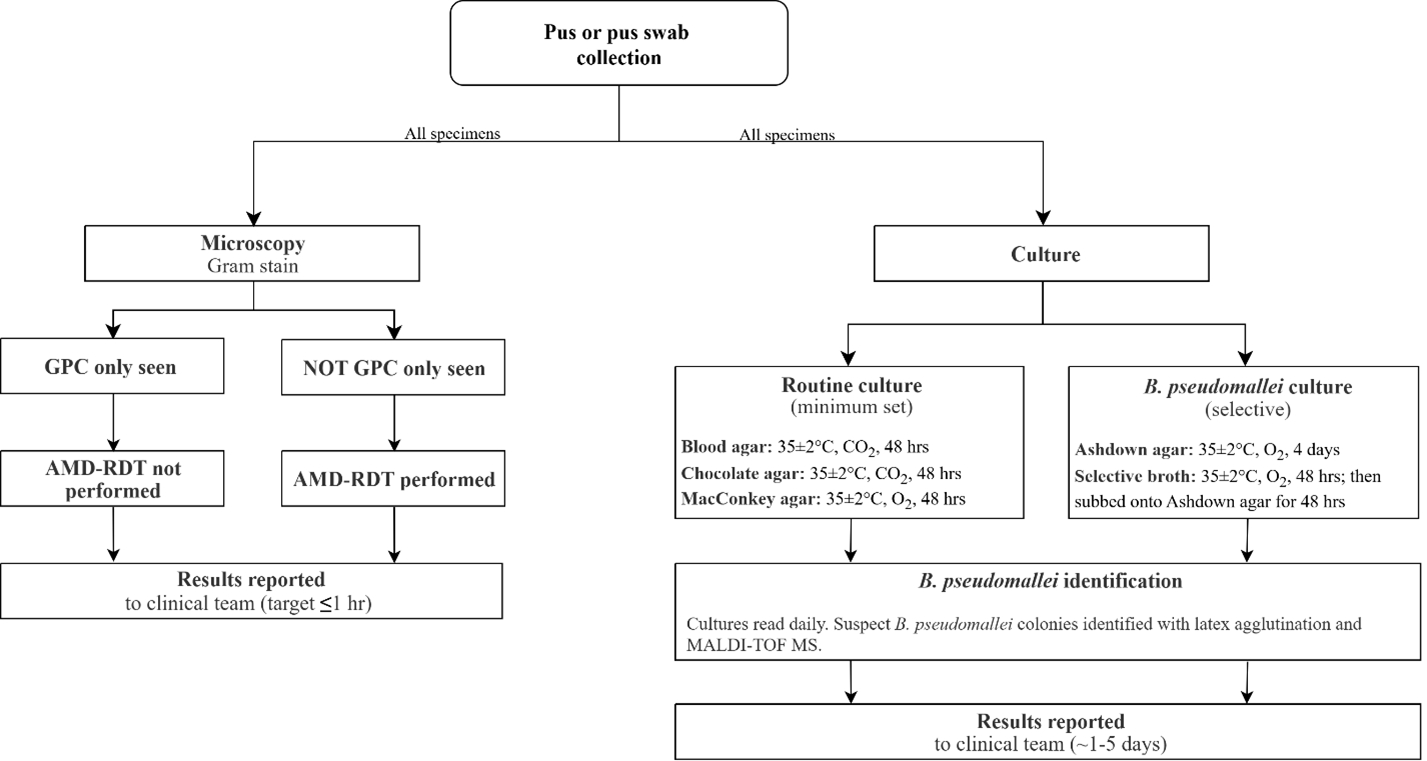

Supplement: S1 Fig — Different media types and incubation conditions were used to allow detection of a broad range of pathogens with varying culture requirements. GPC: Gram-positive cocci; AMD-RDT: Active Melioidosis Detect Plus rapid diagnostic test; B. pseudomallei: Burkholderia pseudomallei; MALDI-TOF MS: Matrix-assisted laser desorption/ionisation mass spectrometry. (TIF) [file pntd.0013962.s001.tif]

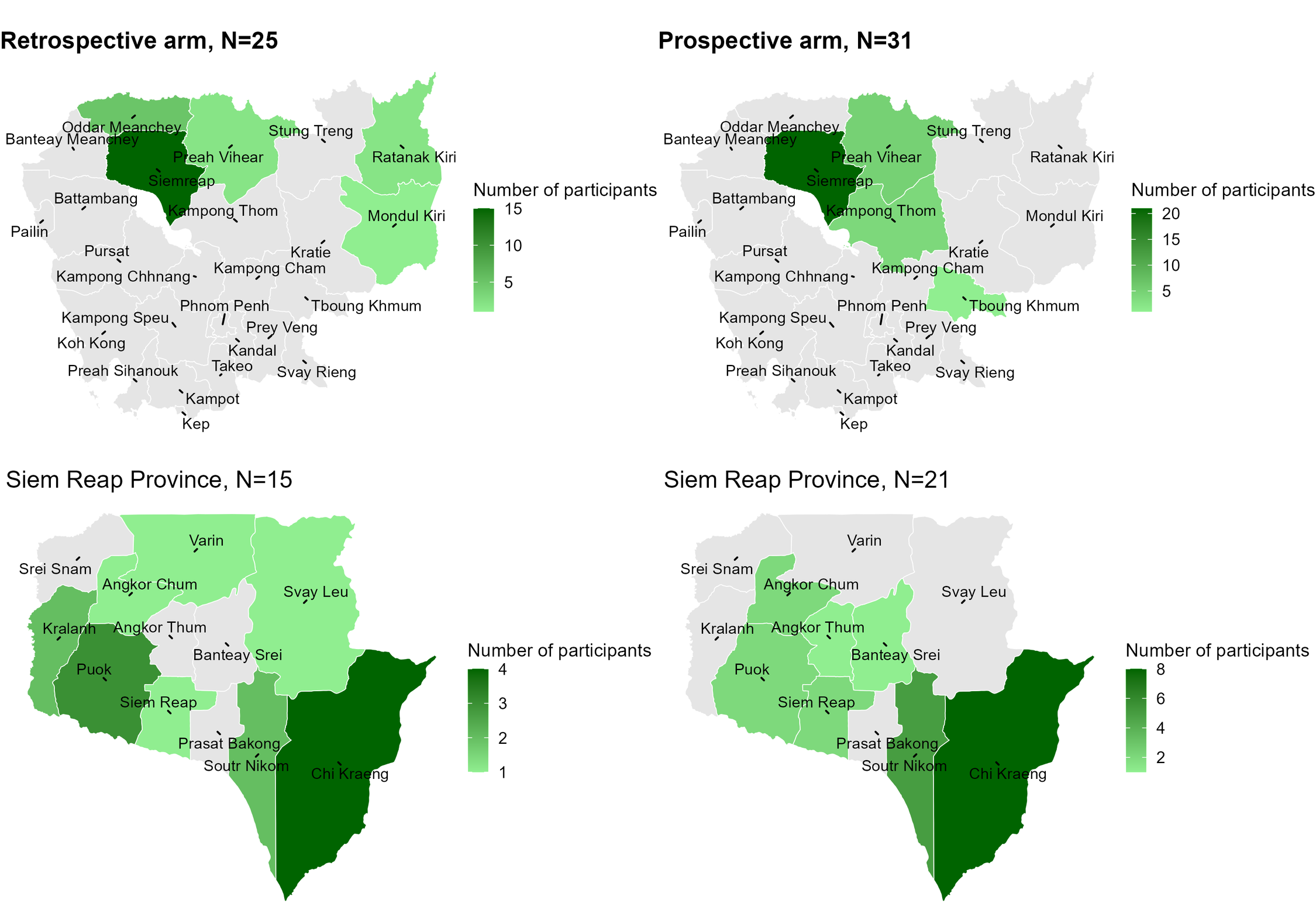

Supplement: S2 Fig — (source of the basemap shapefile: https://data.humdata.org/dataset/cod-ab-khm). (TIF) [file pntd.0013962.s002.tif]

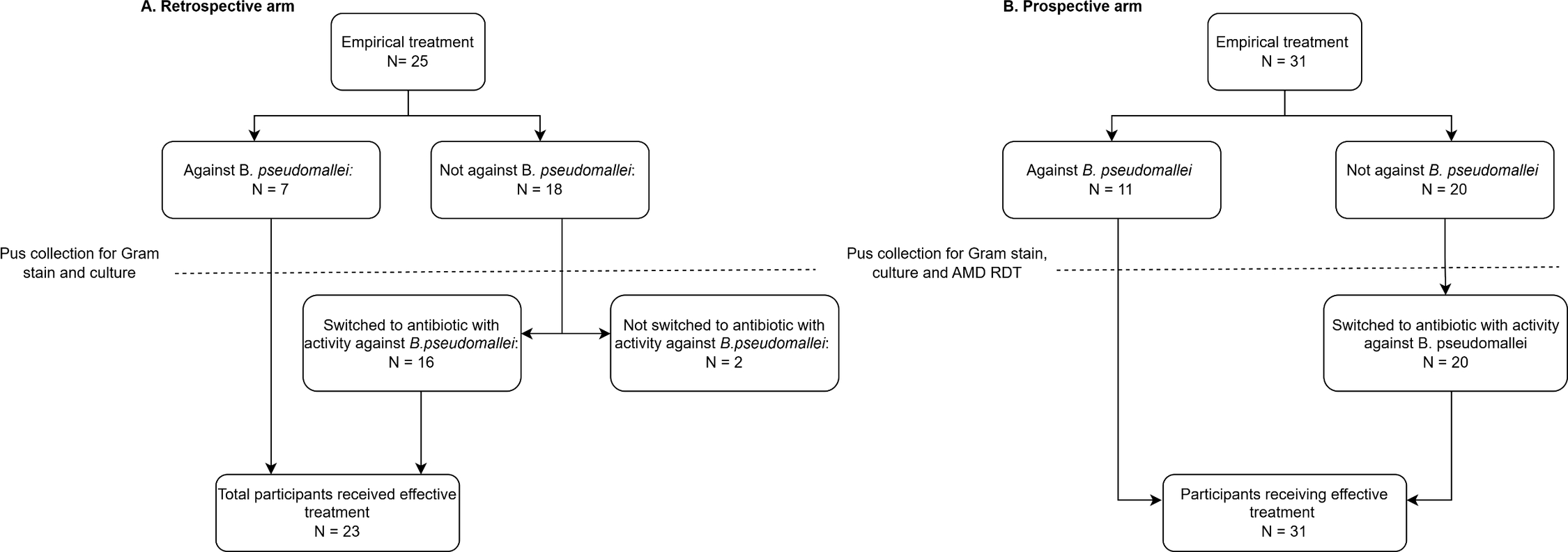

Supplement: S3 Fig — A: Retrospective arm, number of participants = 25; B: Prospective arm, number of participants = 31. (TIF) [file pntd.0013962.s003.tif]
