# Supplementary material for: Rapid diagnosis of skin and soft tissue melioidosis in children
Source: PLoS Negl Trop Dis. 2026 Feb 3;20(2):e0013962. doi: 10.1371/journal.pntd.0013962 (PMC12880741; doi:10.1371/journal.pntd.0013962)
Supplement: S1 Text. — Summary of information reported in accordance with CONSORT 2010 checklist (source of the checklist: https://www.equator-network.org/reporting-guidelines/consort-2010-statement-extension-to-randomised-pilot-and-feasibility-trials/). (DOCX) [file pntd.0013962.s004.docx]

**S1 Text. Summary of information reported in accordance with CONSORT 2010 checklist for feasibility study**

| Section/Topic | Item No | Checklist item | Reported on page No |
| --- | --- | --- | --- |
| Title and abstract | | | |
|  | 1b | Structured summary of study design, methods, results, and conclusions (for specific guidance see CONSORT abstract extension for pilot trials) | 2 |
| Introduction | | | |
| Background and objectives | 2a | Scientific background and explanation of rationale and reasons for the study | 3, 4 |
|  | 2b | Specific objectives or research questions for the study | 5 |
| Methods | | | |
| Trial design | 3a | Description of study design | 5, |
| Participants | 4a | Eligibility criteria for participants | 5, 6 |
|  | 4b | Settings and locations where the data were collected | 5, 6 |
|  | 4c | How participants were identified and consented | 5, 6 |
| Interventions | 5 | The interventions with sufficient details to allow replication, including how and when they were actually administered | 7, 8 |
| Outcomes | 6a | Completely defined prespecified assessments or measurements to address each objective specified in 2b, including how and when they were assessed | 9 |
|  | 6b | Any changes to the study assessments or measurements after the study commenced, with reasons | 9 |
| Sample size | 7a | Rationale for numbers in the study | 9 |
| Implementation | 10 | Who enrolled participants, and who assigned participants to interventions | 5, 6 |
| Blinding | 11b | If relevant, description of the similarity of interventions | 5, 6 |
| Statistical methods | 12 | Methods used to address the study objective whether qualitative or quantitative | 10 |
| Results | | | |
| Participant flow (a diagram is strongly recommended) | 13a | For each group, the numbers of participants who were approached and/or assessed for eligibility, received intended intervention, and were assessed for each objective | 11 |
|  | 13b | For each group, losses and exclusions after assessed for eligibility, together with reasons | 11 |
| Recruitment | 14a | Dates defining the periods of recruitment and follow-up | 10, 14 |
| Baseline data | 15 | A table showing baseline demographic and clinical characteristics for each group | 12, 13 |
| Numbers analysed | 16 | For each objective, number of participants (denominator) included in each analysis. If relevant, these numbers  should be by before and after intervention group | 10, 11, 14 |
| Outcomes and estimation | 17 | For each objective, results including expressions of uncertainty (such as 95% confidence interval) for any  estimates. If relevant, these results should be by before and after intervention group | 15, 16, 17, 18 |
| Ancillary analyses | 18 | Results of any other analyses performed | 12, 13 |
| Harms | 19 | All important harms or unintended effects in each group (for specific guidance see CONSORT for harms) | NA |
|  | 19a | If relevant, other important unintended consequences | NA |
| Discussion | | | |
| Limitations | 20 | Study limitations, addressing sources of potential bias and remaining knowledge gap if any | 20 |
| Generalisability | 21 | Generalisability (applicability) of diagnostic test and findings to standard of care and other studies | 19, 20 |
| Interpretation | 22 | Interpretation consistent with study objectives and findings, balancing potential benefits and harms, and considering other relevant evidence | 18, 19, 20 |
| Other information | | |  |
| Funding | 25 | Sources of funding and other support (such as supply of drugs), role of funders | 21 |
|  | 26 | Ethical approval or approval by research review committee, confirmed with reference number | 6 |
