# Supplementary material for: Rapid diagnosis of skin and soft tissue melioidosis in children
Source: PLoS Negl Trop Dis. 2026 Feb 3;20(2):e0013962. doi: 10.1371/journal.pntd.0013962 (PMC12880741; doi:10.1371/journal.pntd.0013962)
Supplement: S2 Text — Selective Broth, B: Ashdown Agar. (DOCX) [file pntd.0013962.s005.docx]

**S2 Text: In-house Media Preparation**

The two in-house culture media preparation methods below were adapted from previously established methodologies [1,2].

1. **Selective Broth**

Reagents

- Colistin, equivalent to 150 mg (e.g., Millimed Colistin-150)
- Tryptone soya broth powder (Oxoid CM 0129)
- Glycerol (Sigma G5516-500ML)
- Crystal Violet 0.1 % (0.1 g/100 mL)

Note: new stock needs to be incubated at 37°C for 2 weeks before use

- Distilled water

Procedure

Preparing Stock Solution

1. Add 3.0 ml of distilled water to one vial of Millimed Colistin-150 (containing colistimethate sodium equivalent to Colistin 150 mg).
2. Mix until the colistimethate (white powder) has fully dissolved to create a 50 mg/mL colistimethate stock solution. Dispense 0.5 mL aliquots into labelled sterile 1.5 mL Eppendorf tubes.
3. Store at -80°C.

## Preparing Medium

1. Mix the following ingredients in a labelled 1 L Duran bottle.

| **No.** | **Ingredients** | **Amount** | |
| --- | --- | --- | --- |
| 1 | Tryptone soya broth (Oxoid CM 0129) | 5 | g |
| 2 | Glycerol | 20 | mL |
| 3 | Crystal Violet 0.1% | 2.5 | mL |
| 4 | Distilled water | 475 | mL |

1. Place a magnetic stirring bar into the bottle.
2. Place the bottle on a stirring hot plate set at ~100°C and stir until the media is thoroughly mixed (note, undissolved powder will dissolve during the autoclaving process).
3. Loosely place the bottle lid on, but do NOT over tighten it.
4. Place a strip of autoclave tape over the bottle lid.
5. Autoclave the bottle at 121°C for 15 minutes.
6. After the autoclave has finished carefully remove the bottle, mix and then place in a 50°C water bath to allow it to cool to ~50°C (cool enough to hold by hand).
7. Whilst the bottle is cooling, prepare sterile 10 mL plain tubes in a test tube rack.
8. To the cooled broth add 0.5 mL colistimethate stock solution (50 mg/mL) and mix thoroughly.
9. Aseptically dispense 5 mL volumes of the broth into 10 mL sterile plain tubes. Re-cap the tubes and allow them to cool completely.
10. When cool label the tubes and perform quality control testing.
11. Store at 4-8°C.
12. **Ashdown Agar**

Reagents

- Tryptone soya broth (Oxoid CM0129)
- Agar No 1 (Oxoid LP0011)
- Glycerol
- Crystal Violet 0.1% (0.1 g/100 mL)

Note: New stock needs to be incubated at 37°C for 2 weeks before use.

- Neutral red 1% (1 g/100 mL; BDH 340564A)
- Distilled water

**Procedure**

1. Place the following ingredients in a labelled 1 L Duran bottle.

| No. | Ingredients | Amount | Unit |
| --- | --- | --- | --- |
| 1 | Tryptone soya broth (Oxoid CM0129) | 5 | g |
| 2 | Agar No 1 (Oxoid LP0011) | 7.5 | g |
| 3 | Glycerol | 20 | mL |
| 4 | Crystal Violet 0.1% (0.1 g/100 mL) | 2.5 | mL |
| 5 | Neutral red 1% (1 g/100 mL; BDH 340564A) | 2.5 | mL |
| 6 | Distilled water | 475 | mL |

1. Place a magnetic stirring bar into the bottle and stir.
2. Place the bottle on a stirring hot plate set at ~100°C and stir until the media is thoroughly mixed (note, undissolved powder will dissolve during the autoclaving process).
3. Loosely place the bottle lid on, but do NOT over tighten it.
4. Place a strip of autoclave tape over the bottle lid.
5. Autoclave at 121°C for 15 minutes.
6. After the autoclave has finished carefully remove the bottle, mix and then place in a 50°C water bath to allow it to cool to ~50°C (cool enough to hold by hand).
7. To the cooled media, add gentamicin for injection (80 mg/2 mL) to give the desired concentration: 62.5 μl (5 mg/L) and mix thoroughly.
8. Pour 20 mL for each plate aseptically and leave overnight (this can be inside a biosafety cabinet)*.*
9. Perform quality control testing and store at 4-8°C.

**References:**

1. Wuthiekanun V, Dance DA, Wattanagoon Y, Supputtamongkol Y, Chaowagul W, White NJ. The use of selective media for the isolation of *Pseudomonas pseudomallei* in clinical practice. J Med Microbiol. 1990 Oct;33(2):121-6. doi:10.1099/00222615-33-2-121.
2. Ashdown LR. An improved screening technique for isolation of *Pseudomonas pseudomallei* from clinical specimens. Pathology. 1979 Apr;11(2):293–7. doi:10.3109/00313027909061954.
