# Supplementary material for: Rapid diagnosis of skin and soft tissue melioidosis in children
Source: PLoS Negl Trop Dis. 2026 Feb 3;20(2):e0013962. doi: 10.1371/journal.pntd.0013962 (PMC12880741; doi:10.1371/journal.pntd.0013962)
Supplement: S1 Table — (DOCX) [file pntd.0013962.s006.docx]

**S1 Table. Characteristics of participants in prospective arm**

| **Characteristics** | **Overall**  **N (%), Total=107** | **Bps culture (+)**  **N (%), Total=31** | **Bps culture (-)**  **N (%), Total=76** |
| --- | --- | --- | --- |
| **Median age** (IQR), years | 5.6 (3.3-8.5) | 3.9 (3.6-6.8) | 6.4 (3.1-9.4) |
| **Sex**  Female  Male | 53 (49.5)  54 (50.5) | 18 (58.1)  13 (41.9) | 35(46.1)  41(53.9) |
| **Clinical presentation**  Parotitis  Skin infection  Lymphadenitis  Other | 22 (20.6)  23 (21.5)  60 (56.1)  2 (1.8) | 15 (48.4)  4 (12.9)  12 (38.7)  0 | 6 (7.9)  20 (26.3)  48 (63.2)  2 (2.6) |
| **Gram stain results**  No bacteria seen  Gram-negative bacilli only  Other/mixed | 69 (64.5)  23 (21.5)  15 (14.0) | 10 (32.3)  19 (61.3)  2 (6.5) | 59 (77.6)  4 (5.3)  13 (17.1) |
| **Empirical treatment**  Against *Bps*  Not against *Bps* | 15 (14.0)  92 (86.0) | 11 (35.5)  20 (64.5) | 4 (5.3)  72 (94.7) |

Numbers are n (%), unless otherwise specified**.** Bps: *Burkholderia pseudomallei;* IQR: interquartile range
