# Supplementary material for: Rapid diagnosis of skin and soft tissue melioidosis in children
Source: PLoS Negl Trop Dis. 2026 Feb 3;20(2):e0013962. doi: 10.1371/journal.pntd.0013962 (PMC12880741; doi:10.1371/journal.pntd.0013962)
Supplement: Attachment — Submitted filename: ROMEO_Response to Reviewers.docx [file pntd.0013962.s008.docx]

Gram stain of *Burkholderia pseudomallei* isolated from a child with skin abscess, demonstrating characteristic **bipolar ('safety-pin') staining**. Image captured following 24 hours of incubation on 5% sheep blood agar
